# An Expressive Bodily Movement Repertoire for Marimba Performance, Revealed through Observers' Laban Effort-Shape Analyses, and Allied Musical Features: Two Case Studies

**DOI:** 10.3389/fpsyg.2016.01211

**Published:** 2016-08-31

**Authors:** Mary C. Broughton, Jane W. Davidson

**Affiliations:** ^1^School of Music, The University of QueenslandBrisbane, QLD, Australia; ^2^Melbourne Conservatorium of Music, The University of MelbourneMelbourne, VIC, Australia

**Keywords:** bodily movement, music performance, Laban effort-shape analysis, audio-visual perception, embodied cognition

## Abstract

Musicians' expressive bodily movements can influence observers' perception of performance. Furthermore, individual differences in observers' music and motor expertise can shape how they perceive and respond to music performance. However, few studies have investigated the bodily movements that different observers of music performance perceive as expressive, in order to understand how they might relate to the music being produced, and the particular instrument type. In this paper, we focus on marimba performance through two case studies—one solo and one collaborative context. This study aims to investigate the existence of a core repertoire of marimba performance expressive bodily movements, identify key music-related features associated with the core repertoire, and explore how observers' perception of expressive bodily movements might vary according to individual differences in their music and motor expertise. Of the six professional musicians who observed and analyzed the marimba performances, three were percussionists and experienced marimba players. Following training, observers implemented the Laban effort-shape movement analysis system to analyze marimba players' bodily movements that they perceived as expressive in audio-visual recordings of performance. Observations that were agreed by all participants as being the same type of action at the same location in the performance recording were examined in each case study, then across the two studies. A small repertoire of bodily movements emerged that the observers perceived as being expressive. Movements were primarily allied to elements of the music structure, technique, and expressive interpretation, however, these elements appeared to be interactive. A type of body sway movement and more localized sound generating actions were perceived as expressive. These movements co-occurred and also appeared separately. Individual participant data revealed slightly more variety in the types and locations of actions observed, with judges revealing preferences for observing particular types of expressive bodily movements. The particular expressive bodily movements that are produced and perceived in marimba performance appear to be shaped by music-related and sound generating features, musical context, and observer music and motor expertise. With an understanding of bodily movements that are generated and perceived as expressive, embodied music performance training programs might be developed to enhance expressive performer-audience communication.

## Introduction

The task of performing music is inherently physical. The investigation reported here builds on growing interest in understanding the relationship between how music is generated and perceived. It focuses on the marimbist in solo and duo conditions to explore how the body generates musical sound, is used to communicate with co-performers to synchronize the performance and in turn, to communicate with the audience. Specifically, it explores the existence of a core repertoire of expressive bodily movements for marimba performance, and identifies key music-related features associated with specific bodily movements. In addition, it explores how audience members' individual music experiences might shape their interpretation of performer movements, and thus influence communicative processes. Laban effort-shape analysis is used as the analytical framework.

Previous research reveals the vital role of body movement in music performance. Vocal performers' bodily movements and facial expressions communicate affective states, head nods regulate the flow of performance, and manual and arm gestures illustrate the music or text, or stand as emblems, as in spoken communication (Davidson, 2001, 2006; Davidson and Coulam, 2006; King and Ginsborg, 2011). Conductors' bodily movements and facial expressions also communicate and signify affective and practical information, such as entrances and exits, tempo and meter, dynamics, and character of the music (Durrant, 1994; Fuelberth, 2003; Maruyama and Thelen, 2004; Wöllner, 2008; Wöllner et al., 2012). In addition, conductors' arm and hand movements are central to ensemble synchronization (Luck and Toiviainen, 2006; Luck and Sloboda, 2007, 2009). Vocalists' and conductors' non-verbal displays can also affect observers', as audience members, judgments of the performer and performance (Van Weelden, 2002; Kurosawa and Davidson, 2005; Wöllner and Auhagen, 2008). While vocalists and conductors are both relatively free to use their hands and arms for expression and communication, instrumentalists are relatively restricted. That is, the range of manual and arm gestures that instrumentalists can make to communicate with each other and the audience is relatively restricted by the physical requirements for sound production.

The bodily movements that the instrumental musician makes as she/he performs vary according to the performer's expressive goals. They also influence audience perceptions of performance. For instance, performing intentionally with different levels of expression, such as deadpan (minimizing all expression), with typical, projected expression and with exaggerated expression results in different movement patterns, and these influence observer judgments of performance expression (Davidson, 1993, 1994; Broughton and Stevens, 2009). Performing musicians' bodily movements can also influence judgments of musical elements, such as note duration (Schutz and Lipscomb, 2007), phrasing and affective tension (Vines et al., 2006). While these studies are examples of how performing musicians' bodily movements affect observers' cognitive, perceptual, and affective judgments, other studies have focused on analyzing musicians' expressive bodily movements in an effort to understand relations between the music being performed and the patterns of bodily movements generated by the performer.

Patterns of bodily movement have been reported as relating to music structure, technical or anatomical constraints, as well as expressive interpretation. For example, Wanderley et al. (2005) reported that clarinetists exhibited typical movement patterns associated with phrasing or metrical considerations pertinent the music being performed. Davidson (2002) noted how structural elements, such as cadence points or phrase peaks, could elicit specific and identifiable expressive movements by a pianist. The biomechanical constraints associated with producing sound (Bejjani and Halpern, 1989), or executing technically demanding passages (Wanderley et al., 2005) have also been reported as concerns shaping the occurrence and form of bodily movements. Additionally, a performer's expressive intention for the music can affect the amplitude of gestures at various musically or structurally important locations in the performance (e.g., Davidson, 1994; Wanderley, 2002; Shoda and Adachi, 2012). Aside from observations of general relationships between expressive movement and sound production, relatively few studies have investigated either whether there exists a core repertoire of expressive bodily movements for a particular instrument type, or the nature of relationships between particular movements in that repertoire and features of the music being performed.

Why might it be useful to identify a core repertoire of expressive movements and associations between particular movements and music-related features? It could be that musicians' effective expressive communication and performance may be enhanced with a detailed understanding of how the movements that they enact are perceived as expressive. Precedents in western art music history demonstrate that during the seventeenth and eighteenth centuries elaborate codes of physical gestures were learned and used to optimize communication with the goal of moving the affections of the audience (Bulwer, 1644; Quantz, 1752). Similarly, in Indian classical music, there is an elaborate series of postures and gestures that communicate a range of expressive information in a feedback loop between performer and audience (Clayton, 2007). In the current study, the aim is to understand how musicians' expressive bodily movements are produced and perceived by exploring links between performer, music and audience through analysis of observational data. Therefore, a practice-led performative position is central to the research (see also Chaffin and Imreh, 2002; Ginsborg et al., 2006; Ginsborg and Chaffin, 2011; Barrett et al., 2014; Broughton and Davidson, 2014). The performative research paradigm centralizes practice in the research and incorporates a variety of methodological approaches and techniques, which can include observation, reflection and interpretation or analysis based on personal experience (Haseman, 2006). Through this paradigm, a range of different data can be generated. Detailed understandings deriving from such research might usefully be applied in evidence-based music teaching, performance practice, and training. This study stems from and is informed by, the first author's professional experience as a percussionist and marimba player.

Relatively few studies have sought to identify a core repertoire of expressive movements for a particular instrument type and to relate that repertoire to the music that is being performed. For example, Clarke and Davidson (1998) undertook a case study of a pianist noting her use of four distinct types of expressive movements: a head nod, a head shake, an upper body “wiggle,” and a forward-backward surge involving the torso and head. In addition to the four distinct types of movements, a regular body sway movement was identified. Expressive hand gestures were noted as present, but not discussed explicitly due to the correspondence with head gestures. The four distinct movement types reported varied in speed and size at different locations across two performances. Some distinct expressive movements were noted at locations consistent across the two performances, such as head nods or shakes leading from one bar into the next, possibly allied to phrase peak/resolution or metric structure. Distinct expressive movements were also noted in sequence and leading to melodic climax, and others associated with the final cadential sequence. The authors did not describe how the other types of expressive movements related to the music performed, but they did note that while some locations of expressive movements were similar across the two performances, there were also locations specific to one performance and not the other.

In another study investigating a pianists' repertoire of expressive bodily movements, Davidson (2007) noted that the hands and head, and to a degree the torso, exhibited distinct movement patterns. Similar head nodding and shaking, upper body “wriggling,” and the body sway, or swinging movements reported by Clarke and Davidson (1998) were observed. Additionally, Davidson noted expressive movements of the left hand, such as hand lifts, lowering below the keyboard line, flicks, arching, and rotations among others. Also again, while the locations of expressive movements were similar across differently intentioned performances, the specific type of movements observed at these locations could differ. In sum, the pianist did appear to have a core expressive movement repertoire that he could draw on somewhat flexibly to perform in different manners and at different times. This repertoire was linked to music structure, as indicated in the notated score, in moments such as cadence points or where there was “space” for individual musical interpretation, such as during notated rests.

Core movement repertoires specific to woodwind players have also been documented. For example, clarinetists have been reported to display expressive movement patterns that include lowering and raising the bell of the instrument in fast upward or slow continuous manners, and other postural adjustments (Wanderley, 2002). However, while some general similarities in expressive movement patterns have been identified, different performers also move in individual styles. For example, Wanderley et al. (2005) noted that some clarinetists preferred to engage in large movements at phrase endings and remain quite still throughout, whereas others moved with greater regularity and fluidity during phrases. Furthermore, some predominantly moved their heads and others bent their knees. The performers' characteristic movement patterns enabled them to be categorized as primarily phrase- or metric-oriented movers. Davidson (2012) also reported that clarinetists' expressive movement patterns included circling and lifting the bell of their instrument, as well as circling with the elbows, and a sideways body sway. This study involved flutists as well as clarinetists. As players of the two different instrument types from the same family (woodwinds), they were observed making similar movements. These included toe taps, elbow circles, circling the end of the instrument, knee bends, torso rising, and head nods. For woodwind players, there too appears to be a core repertoire of expressive movements that can be deployed in a flexible manner according to music structure, technical or biomechanical constraints, and individual expressive interpretation. However, while there appears to be some commonality in instrument-specific expressive movement patterns, an individual performer's core repertoire might demonstrate preferences for certain types of movements, as well as some idiosyncratic movements.

Studies have predominantly examined expressive bodily movement repertoires in solo performance contexts. How a core movement repertoire employed in a collaborative music making context might differ from a solo context is largely unknown. In what is to our knowledge the only study making such a comparison, Davidson (2012) reported that although the movement types observed across solo and duo performances were largely similar, flutists and clarinetists modified their expressive movements from a solo performance context to synchronize and interact with a co-performer in a duo performance context. Williamon and Davidson (2002) similarly observed that through the rehearsal process, duo pianists increasingly coordinated their non-verbal behaviors, and especially so at locations deemed “important” to generate a tightly coordinated performance and communicate expressive ideas. The swaying type of bodily movement noted in previous studies of solo pianists (Clarke and Davidson, 1998; Davidson, 2007) has also been observed in duo piano (Williamon and Davidson, 2002) and duo woodwind performances (Davidson, 2012). In a study of duo pianists that involved tracking the performers' movements while playing, Keller and Appel (2010) highlighted the importance of body sway in ensemble synchronization. Performance was best synchronized when the pianists' body sway (anterior-posterior) was coordinated. Although visual contact was not a prerequisite for synchronized performance, without visual contact, the size of the body sway movement increased. In addition, the patterning of body sway and synchronization revealed leader-follower relations. The extant literature suggests that a musicians' repertoire of bodily movements made in solo performance is also present, in some form, in a collaborative music making context. Looking across different instruments, body sway appears to be an important component of a core expressive bodily movement repertoire.

The present study adds to the literature by examining marimba playing in solo and duo performance contexts. This study complements existing work that has used a number of techniques and analytic frameworks: researchers as observers, quantitative kinematic measures, and idiosyncratic descriptive categorization and coding systems. In an effort to minimize potential researcher-analyst bias, our data are based on observational analyses of the bodily movements perceived as expressive by independent observer-analysts. Furthermore, recent research suggests that individual differences in observers' specialist motor expertise influences their perception of, and cognitive judgments regarding performing musicians' bodily movements (Wöllner and Cañal-Bruland, 2010; Broughton and Stevens, 2012; Broughton and Davidson, 2014). Therefore, the present study employs six observers across two case studies. The observers are all professional musicians; three are percussionists and experienced marimba players. Secondly, the core expressive movement repertoire for marimba playing is based on observers' perception of expressive bodily movement, rather than all the movement evident in performance. This approach is underpinned by previous research, which demonstrates that observer perception of expressiveness is not distributed evenly throughout performance, and moments of high and low amplitude movements can both be perceived as expressive (Davidson, 1994, 2002). Finally, our data are derived using an analytical framework that can be applied to study different instrumental/vocal contexts and performers, that is Laban effort-shape analysis. This analytical framework has been used previously in studies of marimba players' expressive bodily movements (Broughton and Stevens, 2012; Broughton and Davidson, 2014).

The use of Laban effort-shape analysis as an observational framework is underpinned by three guiding principles. Firstly, effort-shape analysis provides a framework for analyzing and describing musicians' expressive bodily movement based on movement principles. This contrasts with most existing research, which has drawn on methods of categorizing non-verbal information found in speech-situated interpersonal communication. In those contexts, manual gestures, facial expressions, eye gaze behaviors, and body postures are in focus and often related to verbal content (e.g., Ekman and Friesen, 1969; Argyle, 1988; McNeill, 1992; Goldin-Meadow, 2003; Kendon, 2004). Secondly, in using effort-shape analysis to analyze performing musicians' expressive bodily movements, we present a present a framework that is potentially applicable across all modes of music performance be it instrumental, vocal or conducted performance, in solo and collaborative music making contexts. Finally, the effort-shape analytical framework offers an expressive movement meta-language that could be used in observational studies and by musicians as they prepare for expressive performance. Musicians could use it as a tool to conceive and embed expression in a performance movement plan to optimize expressive communication with an audience. This use of effort-shape analysis as a shared tool with which expressive bodily movements are conceived and documented by musicians and analyzed by observers, offers a further opportunity to undertake a detailed examination of the meeting point between performance, practice-based and practice-led research.

## Laban effort-shape analysis

Laban effort-shape analysis is a systematic approach to analyzing and understanding expressive bodily behavior. The approach draws on Laban Movement Analysis (LMA) concepts and analytical techniques (Laban, 1988). Underpinning LMA is the principle that bodily behavior reflects an individual's inner motivation for movement (Bartenieff and Lewis, 1980). Although the LMA approach has its origins in Rudolph Laban's (1879-1958) work in dance, dance notation (*Labanotation*) and human movement (Laban, 1988), it has been employed to analyse and understand human movement behavior in a range of contexts. These include clinical settings (Higgins, 1993; Foroud and Whishaw, 2006) through to music performance (Broughton and Stevens, 2012; Broughton and Davidson, 2014) and anthropology (Jablonko and Kagan, 1988). The LMA approach assesses four main components of bodily movement. A degree of *effort* is required for the *body* to move through *space*; the body makes various *shapes* as it moves through space (Bartenieff and Lewis, 1980). Effort-shape analysis focuses on the effort and shape components of the LMA approach. The focus of effort-shape analysis is to capture the expressive qualities inherent in bodily movement and document *how*, rather than what movement occurs (Davis, 1970).

The LMA observational analysis techniques, which are also employed in effort-shape analysis, involve a combination of visual inspection and kinesthetic mirroring processes to understand how expressive bodily action both looks and feels to perform. Kinesthetic mirroring and introspection assist the observer to analyze and categorize the bodily behavior according to the framework. Here we offer a brief overview of the effort-shape analytical system. More detailed descriptions can be found in previous published work (Broughton and Stevens, 2012; Broughton and Davidson, 2014). Effort refers to the recognizable patterns of tension, release and phrasing of physical exertion evident in expressive bodily movement (Maletic, 1987). Shape refers to the way the body takes shape in space and is conceptually interconnected with effort (Royce, 2002). A whole bodily involvement in expressive action reflects *postural effort*, whereas movement of only the body part required to perform the job reflects *gestural effort* (Lamb and Watson, 1979; Bartenieff and Lewis, 1980). In the next section, we outline the components of effort analysis, followed by shape.

Effort may be “goal-directed” or “non-goal-directed” (Bartenieff and Lewis, 1980). “Goal-directed” actions are basic working actions that function directly to achieve a functional goal, such as dabbing with a paint brush, or gliding an iron over silk cloth. These “goal-directed” expressive actions create particular qualities of movement and are defined descriptively, or metaphorically. “Non-goal-directed” actions communicate mood or emotional states and are defined metaphorically. In contrast to “goal-directed” actions, these types of movements or actions are not directed toward achieving a particular functional goal. For example, contrast the characteristic movement patterns of a person who is irate about something with those of a person who might be deep in concentration trying to decipher flat-pack furniture instructions.

In analyzing effort, different qualities of bodily expression are revealed through different combinations of *effort elements* associated with *motion factors*. The four motion factors are *weight, time, space*, and *flow*. The effort elements associated with each motion factor are *strong*/*light* weight, *sudden*/*sustained* time, *direct*/*indirect* space, and *bound*/*free* flow. The different combinations of effort elements associated with the motion factors weight, time, and space result in a total of eight *basic effort actions* (see Table 1). These are “goal-directed” basic working actions, featuring two distinct phases—exertion and relaxation (Laban and Lawrence, 1974). When the flow motion factor replaces the weight, time, or space motion factor, the result is a *transformation drive*. Transformation drives reflect “non-goal-directed” action to communicate mood or emotional tone. Such actions typically are of longer duration than bi-phasic basic effort actions. “Spell”-like bodily behavior can give the appearance of someone casting a spell, “Vision” can appear as if the person is concentrating or deep in thought, and “Passion”-like bodily behavior can manifest as gentle caresses or as if the person is gesticulating in a wild rage. The metaphoric name for each basic effort action and transformation drive reflects the visual appearance as well as the kinesthetic sensation of doing the action. Shape is analyzed with regard to the vertical, horizontal, and sagittal axes of spatial movement. Shaping movement can appear as “Rising”/“Sinking” on the vertical axis, “Widening”/“Narrowing” on the horizontal axis, and “Advancing”/“Retreating” on the sagittal axis of space.

**Table 1:** **The metaphoric names associated with the eight basic effort actions, three transformation drives, and shape features**.

**Effort-shape component**	**Metaphoric name**	**Weight**	**Space**	**Time**	**Flow**	**Axis of space**
Basic effort actions	“Punch”	Strong	Direct	Sudden	na	
	“Dab”	Light	Direct	Sudden	na	
	“Press”	Strong	Direct	Sustained	na	
	“Glide”	Light	Direct	Sustained	na	
	“Slash”	Strong	Indirect	Sudden	na	
	“Flick”	Light	Indirect	Sudden	na	
	“Wring”	Strong	Indirect	Sustained	na	
	“Float”	Light	Indirect	Sustained	na	
Transformation drives	“Passion”	Strong/light	na	Sudden/sustained	Bound/free	
	“Spell”	Strong/light	Direct/indirect	na	Bound/free	
	“Vision”	na	Direct/indirect	Sudden/sustained	Bound/free	
Shape	“Rising”					Vertical
	“Sinking”					Vertical
	“Widening”					Horizontal
	“Narrowing”					Horizontal
	“Advancing”					Sagittal
	“Retreating”					Sagittal

*Motion factor and effort element combinations are noted for the effort components of the system; axis of spatial movement is noted for the shape components of the system*.

The analytical process involves first identifying any moments in the performance material that stand out to the observer as expressive in terms of bodily movement or stillness. The observer then uses a combination of visual inspection and introspection regarding the kinesthetic experience of overtly or covertly performing the observed expressive bodily behavior to categorize it as a basic effort action or a transformation drive. Often the metaphoric name is sufficient to perform the effort analysis. Occasionally, the observer will have to engage in a “bottom-up” decision-making process to analyze effort. That is, the observer will need to decide whether the expressive bodily behavior is “goal-directed” or “non-goal-directed.” The particular combination of motion factors and effort elements evident in the movement will then require identification using visual inspection, kinesthetic mirroring, and introspection processes. For instance, shape is analyzed in terms of the degree to which the body's postural movement appears to be involved in the effortful, expressive action—“Rising”/“Sinking,” “Widening”/“Narrowing,” and “Advancing”/“Retreating.”

It should be noted that effort and shaping bodily behaviors can appear very subtle in terms of movement quantity, yet be reliably discerned by observers as expressive in quality (Broughton and Stevens, 2012, also see Broughton and Davidson, 2014 for a series of screen shots of figures illustrating exemplar actions). This finding is supported by studies using different methodologies (behavioral data, observational frameworks, and kinematic measures), leading to the conclusion that perceived expressiveness is constituted by more than quantity of movement. There needs to be a certain expressive quality perceived in bodily movement or stillness for observers to identify a moment in performance as expressive (Davidson, 1994, 2007). Some inter-judge reliability evidence exists for the effort-shape analytical system as described here and applied to music performance contexts (Broughton and Stevens, 2012; Broughton and Davidson, 2014). A few other studies have reported satisfactory inter-judge reliability for effort and shape building blocks (McCoubrey, 1984; Davis, 1987; Du Nann Winter et al., 1989), as well as other movement analysis systems that have incorporated effort and shape components (Davis, 1987; Sossin, 1987).

The principal aims of this study are threefold: (i) investigate the existence of a core repertoire of marimba performance bodily movements that is perceived similarly by different observers as expressive; (ii) identify key music-related features associated with the core repertoire; and (iii) to understand how observers' perception of expressive bodily movements in marimba performance might differ, according to observers' individual differences in music and motor expertise. We address these aims through two observational case studies, using Laban effort-shape analysis as the analytical framework. This study builds on the insights gained through previous studies (Broughton and Stevens, 2012; Broughton and Davidson, 2014) by examining participants' observations of the subcomponents of the basic effort action, transformation drives, and shape feature categories in detail. This in turn builds from a developing use of Laban's work in capturing the expressive qualities and shapes of movement patterns in other areas than dance, for example, robot development (Lourens et al., 2010), everyday bodily movement (Levy and Duke, 2003), and management (Moore, 1982).

## Case study 1: solo marimba performance

### Participants

Participants in Case Study 1 were all professional musicians. A female and a male percussionist, who were also experienced marimba players, a female violinist, and a male French hornist acted as observer participants. Although they did not report their exact ages, the observer participants' approximate ages ranged from mid-twenties to mid-forties.

### Materials

The material for analysis in Case Study 1 was 16 (20–25 s) audio-visual recorded excerpts of solo marimba performance (see Broughton and Stevens, 2012). The excerpts were drawn from the stimuli used in an experiment reported previously (Broughton and Stevens, 2009).

The stimulus material in Broughton and Stevens (2009) comprised 96, 20–25 s excerpts from twentieth-century marimba repertoire: *Marimba Dances, II* and *III* by Edwards (1990); *Suite No.2 for Solo Marimba, I* and *III* by Yoshioka (1995); *Nancy* by Séjourné (1989); and *Merlin* by Thomas (1989). Two professional marimba players (one female; one male), dressed in black, performed the excerpts in two performance manners: *projected* (with an expressive intention consistent with a public performance) and *deadpan* (intentionally minimized expression). The performances were recorded audio-visually using a Panasonic digital video camera (NV-MX300EN/A) with sound recorded through and an external RØDE NT4 stereo condenser microphone and Behringer mixing desk. The digital video camera and microphone were placed directly in front of the instrument and took the marimba's full length and height of the performers into frame. The marimba was a Malletech Stiletto instrument played with Encore Nancy Zeltsman series and Mike Balter mallets. The audio component of the audio-visual excepts was subjected to group normalization processes (using Adobe Audition 2.0) to ensure comparable volume across musicians and performance manners for the musical excerpts. A rectangular opaque box, created from the off-white background in the video, was placed on the screen (using Adobe Premier Pro 1.5) to mask the performers' facial expressions but enable the observer to view the performers' head movements. In Broughton and Stevens (2009), participants were presented with a set of 16 audio-visual excerpts, and a different set of 16 audio-only excerpts (where they viewed a black screen) with sound presented through Koss (UR20) headphones. Full counterbalancing processes in the study design controlled for potential order effects (see Broughton and Stevens, 2009). Participants judged each music excerpt on two separate seven-point Likert scales: *expressiveness* (*very inexpressive-very expressive*) and *interest (very uninterested-very interested*).

The excerpt selection for analysis as Case Study 1 (and as in Broughton and Davidson, 2012) was based on the ratings of expressiveness given by musically trained and untrained participants in Broughton and Stevens (2009). Four highly rated projected performances, and four low-rated deadpan performances were selected. The projected and deadpan selected excerpts were balanced in terms of tempo (fast/slow) and performer (female/male). Additionally, the same excerpts performed by the same marimbist but in the alternative manner were selected for comparative analysis. This made a total of 16 excerpts. Of the 16 excerpts, eight were performed in a projected manner, and the remaining eight excerpts were performed in a deadpan manner. The music excerpts were from: *Suite No. 2 for Solo Marimba, III* by Yoshioka (1995); *Marimba Dances, II and III* by Edwards (1990); *Nancy* by Séjourné (1989); *Merlin, I* by Thomas (1989). The musical material was familiar to the observer percussionists, but unfamiliar to the violinist and hornist. A DVD of the performance recording was provided to participants for playback on television or computer.

### Procedure

Participants gave informed written consent prior to taking part in the research. The research conformed to Australian regulatory standards. The observational research methodology was approved by The University of Western Sydney (now Western Sydney University) Human Research Ethics Committee.

### Observer training: case study 1

All participants were provided a 1-h individual training session in effort-shape analysis, and the particular analysis task. The first author provided the training, as she was experienced in conducting effort-shape analysis as applied to music, and in particular marimba, performance. During the training session, participants were introduced to effort-shape analysis and provided with written reference material (see Broughton and Stevens, 2012). Participants practiced the observational techniques and actions, and were shown audio-visual recordings of marimba playing (from the first-author's library and YouTube; performers were not those featured in the stimulus material) to discuss as illustrative audio-visual examples of the various components of the system. As this was the first study of its kind, this was the most suitable performance material to use for observer training. In conducting their analyses, participants considered the carriage of the body as a whole, focusing on limb, torso, and head movement, as they deemed important to include in their analysis. Participants were not required to document exactly which part of the body their attention was focused on when analyzing the different components. However, it is likely that analyses of basic effort actions focused attention on the working actions of the hands and arms required to produce sound. Shaping features would reflect a spread of effort throughout the body (postural effort), which would be evident in limb, torso, and head movement deviating from a neutral upright standing posture. Transformation drives would be revealed through combinations of limb, torso, and head movement reflecting distinctly different qualities of mood or emotion.

Once participants were satisfied that they understood the effort-shape analytical system and felt confident that they could independently apply the system to analyze the performance material, the individual task and procedure were outlined. They were then given a period of ~1 week to complete the set task at their own convenience. Participants were informed that they were able to start, stop, and replay the performance material as necessary, with and without sound, in order to complete their set task. We expected that permitting participants to analyze the performance material with and without sound would not adversely affect participants' analyses, as previous research shows that musically trained participants judge music performance in audio-visual and vision-only formats similarly (Davidson, 1993).

### Case study 1 tasks

The participants in Case Study 1 performed three different tasks. The percussionists completed a verification task, the violinist an independent analysis task, and the hornist completed a signal-detection-driven yes/no task. The verification task asked percussionist participants to review the performance material in conjunction with music scores annotated with effort-shape analyses of expressive bodily movements observed in the recorded performance material. The task was to agree/disagree with the effort-shape analyses provided. The independent analysis task asked the violinist to independently annotate the music scores provided with her own effort-shape analyses of the performers' bodily movements in moments she perceived as expressive in the allied recorded performance material. The signal-detection yes/no task asked the hornist to accept/reject effort-shape analyses documented on the music scores accompanying the performance material. In this task, 50% of the annotations were those that all the percussionists in the verification task had agreed upon as accurate depictions of their expressive movement observations. An additional 50% of “fake” effort-shape annotations were included as “catch trials,” but at musically plausible locations. The “catch trials” matched the number and type of each sub-component of the system of the “agreed” real annotations, and were randomly distributed across the performance material for analysis. The hornist was informed of the proportion of real and “catch trial” annotations. A detailed report of the procedure and each task that resulted in the data used here as Case Study 1 are reported elsewhere (see Broughton and Stevens, 2012).

### Results

Firstly, the frequency of effort and shape observations per participant were totaled (see Table 2). Analysis then involved pooling the observations where all participants agreed on observing the same type of action at the same location in the performance material.

**Table 2:** **Frequency of effort-shape observations by each participant in the two case studies according to the particular analysis task performed by observer participants**.

**Case study**		**Case Study 1**				**Case Study 2**	
**Task**		**Verification task**	**Independent analysis task**	**Signal-detection yes/no task**		**Independent analysis task**	
**Observer**		**Percussionists**	**Violinist**	**French hornist “verified” (correct identification)**	**French hornist “non-verified” (“catch trial”)**	**Percussionist**	**Vocalist**
Basic effort actions	“Punch”	34	10	34	33	0	0
	“Dab”	40	12	37	36	18	14
	“Float”	17	4	13	16	17	0
	“Glide”	7	8	6	6	1	13
	“Press”	6	0	6	3	0	0
	“Flick”	1	5	1	1	13	0
	“Slash”	0	0	0	0	0	0
	“Wring”	0	0	0	0	0	0
Transformation drives	“Passion”	11	4	11	6	0	0
	“Vision”	17	4	15	16	0	0
	“Spell”	5	11	4	3	3	0
Shape features	“Rising”	67	46	55	20	4	38
	“Sinking”	77	30	64	40	0	14
	“Widening”	25	37	20	19	2	0
	“Narrowing”	0	11	0	0	0	0
	“Advancing”	2	4	2	1	0	0
	“Retreating”	5	2	5	1	1	0

**Figure 1:**
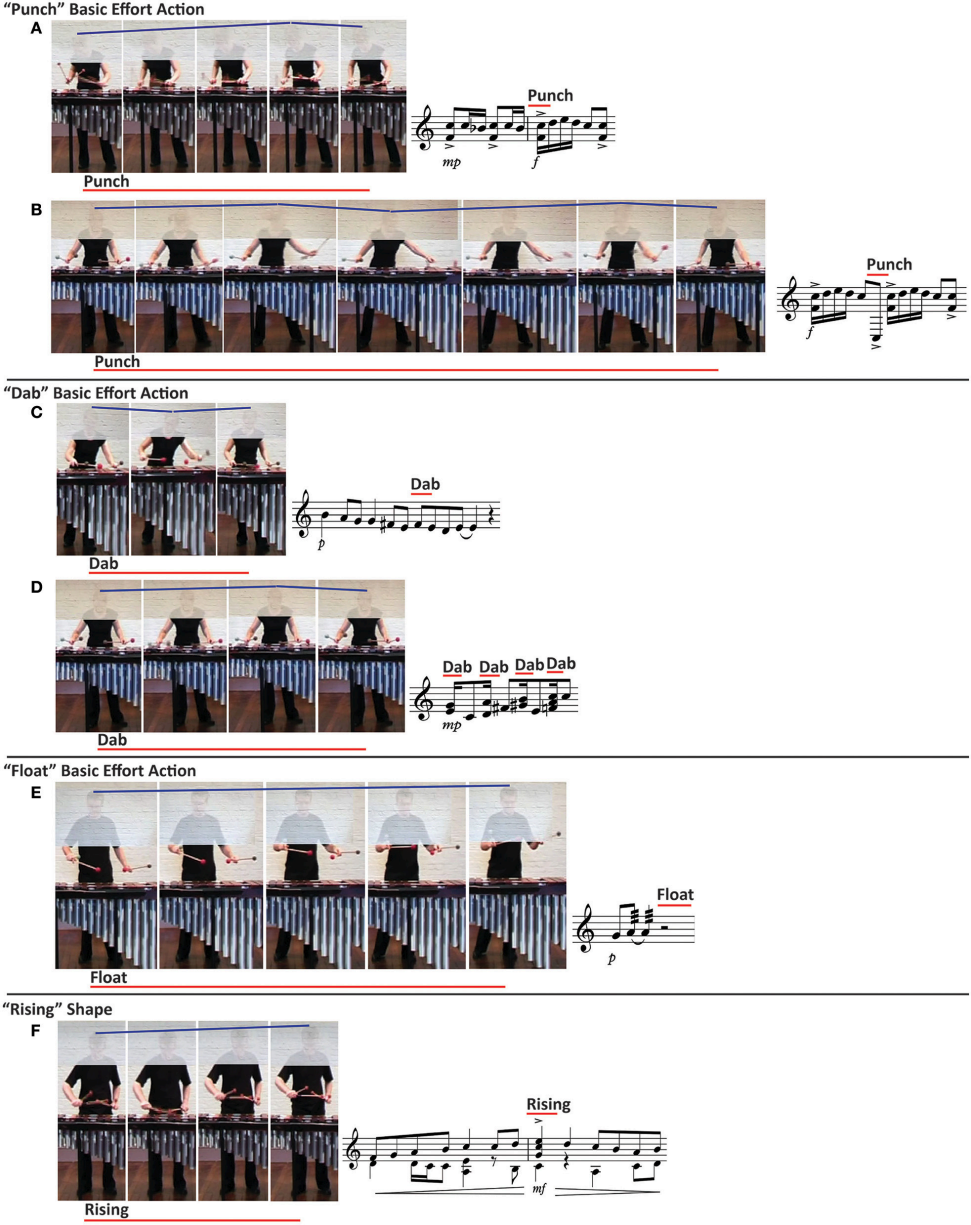
**Effort and shape feature movement examples as illustrated with still images drawn from Case Study 1**. The red line on top of the accompanying musical example illustrates the duration and placement of the movement in relation to the music performed. The number of still images included does not reflect the duration of the movement, simply sufficient images to illustrate each action type. The musical notation accompanying each example is indicative of the allied music excerpt, though not a reproduction from the original music scores. The blue line above the performers' heads indicates the direction of movement on the vertical axis of space. Participants analyzed the carriage of the body as a whole, focusing on limb, torso, and head movement, as they deemed important to include in their analysis. Note: “Punch” actions and “Dab” actions differ in the weight effort element, meaning that they might exhibit some visual similarity (in movement trajectories and temporal profiles), but kinesthetically, and hence qualitatively, are very different. Both “Punch” and “Dab” are comprised of sudden time and direct space. However, “Punch” has strong weight and “Dab” is comprised of light weight. Try performing “Punch” and “Dab” actions, simply as actions and then mimicking the movement shown through the still-image examples, to experience the different kinesthetic sensation between them.

A small repertoire of expressive bodily movements was observed in common by the percussionists, violinist and French hornist at the same points in the performance material. The primary expressive bodily movements observed were “Punch” (*n* = 9), “Dab” (*n* = 2) and “Float” (*n* = 2) basic effort actions, and “Rising” (*n* = 30), “Sinking” (*n* = 21) and “Widening” (*n* = 11) shape features. “Rising” shape features were also observed in conjunction with “Punch,” “Dab,” and “Float” basic effort actions. No “Slash” or “Wring” basic effort actions were observed. “Passion” (*n* = 4), “Vision” (*n* = 3), and “Spell” (*n* = 1) transformation drives were observed at similar locations by the four participants. Figure 1 illustrates some exemplar expressive actions and indicative allied music excerpts (though not original reproductions from the music scores) for Case Study 1.

The percussionists, violinist, and French hornist reported “Punch” basic effort action observations in conjunction with accented notes and *forte* (loud) dynamic markings. “Float” basic effort actions were observed during notated rests, which permitted movement of the hands and mallets away from the instrument. Expressive movement observations were not consistently related to markings in the music score. For example, while “Dab” actions were observed delineating rhythmic grouping music elements (Figure 1D), they were also observed at the center of phrases (Figure 1C). Furthermore, Figure 1A show a series of accent markings in the score but only one was associated with a “Punch” observation. “Punch” actions were also noted where a great distance on the instrument had to be traversed between consecutive notes to maintain the flow and timing of the performance, see Figure 1B. “Punch” and “Dab” actions are qualitatively different, and observers can detect the difference through visual observation and kinesthetic mirroring analytical processes. “Punch” actions and “Dab” actions differ only in the weight effort element, meaning that they might exhibit some visual similarity (in movement trajectories and temporal profiles), but are very different kinesthetically, and hence qualitatively. The four observers also noted “Rising” shape features in relation to phrasing (beginnings, peaks, and ends), rhythmic note groupings and marking the tempo in fast music, and in conjunction with a rising musical line in slow lyrical music. “Sinking” shape features were observed at the beginnings and ends of phrases, or preceding or following “Rising” in rhythmic excerpts, or when marking the tempo (or sub-divisions of the tempo) in slow music. “Widening” shape features were seen at phrase peaks in a moderately slow tempo, lyrical piece.

The “Passion” transformation drive was observed in slow-tempo music of a quiet dynamic where the music required that all four mallets be used (two held in each hand) to play chords and phrase musical lines in the style of a chorale. On the marimba, this necessitates using roll techniques, whereby fast alternating hand movements make the mallet heads contact the instrument bars in quick succession, with the goal of producing the illusion of a sustained sound. This technique helps the performer to dynamically shape musical phrases and lines within phrases. The “Vision” transformation drive was noted in fast-tempo music at a medium dynamic level, and where the music required some fast movement between relatively awkward body positions in order for the mallets to strike the right notes in time. The “Spell” transformation drive was observed at one location in a piece that was of a very quiet dynamic level and a slow tempo. This excerpt required the performer to reach across the widest part of the instrument, in the bass range, to play the specified notes.

Examination of individual participant data revealed differences in the frequency with which the various sub-components of the basic effort action, transformation drive, and shaping feature categories were observed (Table 2). Separate chi-square goodness-of-fit tests conducted on individual participant data revealed significant differences between observational frequencies for different action types within each category. For the percussionists' basic effort action observations, χ^2^(5, *n* = 105) = 73.91, *p* < 0.01, the order of most to least frequent observations was: “Dab” (40, 38.1%), “Punch” (34, 32.38%), “Float” (17, 16.19%), “Glide” (7, 6.67%), “Press” (6, 5.71%), and “Flick” (1, 0.95%). For transformation drives, χ^2^(2, **n** = 33) = 6.55, *p* < 0.05, the order of most to least frequent observations was: “Vision” (17, 51.52%), “Passion” (11, 33.33%), and “Spell” (5, 15.15%). For shape features, χ^2^(4, **n** = 176) = 138.55, *p* < 0.01, the order of most to least frequent observations was: “Sinking” (77, 43.75%), “Rising” (67, 38.07%), “Widening” (25, 14.2%), “Retreating” (5, 2.84%), and “Advancing” (2, 1.14%).

The violinist's frequencies of observations for different action types did not significantly differ within the basic effort action, χ^2^(4, *n* = 39) = 5.74, *p* = 0.22, or transformation drive categories, χ^2^(4, **n** = 19) = 5.16, *p* = 0.08. However, for shape features, a significant difference in observational frequencies was observed, χ^2^(5, *n* = 130) = 78.89, *p* < 0.01. The order of most to least frequent observations was: “Rising” (46, 35.38%), “Widening” (37, 28.46%), “Sinking” (30, 23.08%), “Narrowing” (11, 8.46%), “Advancing” (4, 3.08%), and “Retreating” (2, 1.54%).

For the French horn player, we conducted two separate chi-square goodness-of-fit tests for his correct identification of the annotations agreed upon by the percussionists in the verification task (“verified”), and incorrect identification of “catch trial” annotations as true observations (“non-verified”). As a reminder, half of the annotations were those expressive movements that the percussionists agreed observing in the verification task. The other half were “catch trials,” or “fake” annotations, matching the number and type of the percussionists' “agreed” annotations, randomly distributed at musically plausible locations on the music scores accompanying performance material for analysis. A significant difference in observational frequencies was found for the French hornist's “verified” observations, χ^2^(5, **n** = 97) = 74.16, *p* < 0.01. The order of most to least frequent observations was: “Dab” (37, 38.14%), “Punch” (34, 35.05%), “Float” (13, 13.4%), “Glide” (6, 6.19%), and “Press” (6, 6.19%) in fourth place, and “Flick” (1, 1.03%). A significant difference was also found in observational frequencies for the French hornist's “non-verified” observations, χ^2^(5, **n** = 95) = 74.71, *p* < 0.01. The order of most to least frequent observations was: “Dab” (36, 37.89%), “Punch” (33, 34.74%), “Float” (16, 16.84%), “Glide” (6, 6.32%), “Press” (3, 3.16%), and “Flick” (1, 1.05%). For transformation drives, a significant difference was found for the French hornist's “verified,” χ^2^(2, **n** = 30) = 6.2, *p* < 0.05, and “non-verified,” χ^2^(2, **n** = 25) = 11.12, *p* < 0.01, observations. The order of most to least frequent “verified” observations was: “Vision” (15, 50%), “Passion” (11, 36.67%), and “Spell” (13.33%). This order was the same for “non-verified” observations: “Vision” (16, 64%), “Passion” (6, 24%), and “Spell” (3, 12%). Finally, for shape features, a significant difference was found for the French hornist's “verified,” χ^2^(4, **n** = 146) = 112.56, *p* < 0.01, and “non-verified,” χ^2^(4, **n** = 81) = 64.86, *p* < 0.01, observations. The order of most to least frequent “verified” observations was: “Sinking” (64, 43.84%), “Rising” (55, 37.67%), “Widening” (20, 13.7%), “Retreating” (5, 3.42%), and “Advancing” (2, 1.37%). This order was virtually the same for “non-verified” observations, except that “Retreating” and “Advancing” tied in last place: “Sinking” (40, 49.38%), “Rising” (20, 24.69%), “Widening” (19, 23.46%), “Retreating” (1, 1.23%), and “Advancing” (1, 1.23%).

### Discussion

A small repertoire of expressive bodily movements was identified from the percussionists, violinist, and French hornist participants' observational effort-shape analyses. This repertoire included a subset of “goal-directed” basic effort actions, “non-goal-directed” transformation drives, and a subset of shaping features. Beyond the core repertoire observed in common, individual differences in participants' motor expertise appeared to shape their observations (Broughton and Stevens, 2012; Broughton and Davidson, 2014). Especially, the violinists' effort-shape observations appeared to diverge from those of the percussionists and French hornist.

The expressive movements observed were associated with elements of the music structure, technical constraints, and expressive interpretation. The results of this study indicated that basic effort actions were primarily linked to features identifiable through the music score, such as rhythmic note groupings and marking the tempo, rests, phrasing, and accent marks (Davidson, 2002, 2007; Wanderley, 2002; Wanderley et al., 2005). However, relationships between certain markings evident in the score and particular actions were not consistent. The technical demands associated with playing a particular piece of music also appeared to influence whether a basic effort action occurred, as well as the type of action (e.g., Bejjani and Halpern, 1989; Thompson and Luck, 2012). For example, “Punch” actions were noted where a great distance on the instrument had to be traversed between sequential notes to maintain the flow and timing of the performance. The increase in the size and speed of movement that is necessary to play the stipulated notes in time at such points in the performance, which could be measured in terms of movement amplitude and velocity, might have influenced observers' perception of these moments as being notably more expressive than at other points (Davidson, 1993, 1994; Juchniewicz, 2008; Broughton and Stevens, 2009; Huang and Krumhansl, 2011). A further consideration is that musical interpretation might provide an additional layer driving deviations from simple systematic associations between musical markings and expressive movements (e.g., Wanderley, 2002; Thompson et al., 2005; Davidson, 2007). In this sense, the music structure, technique, and expressive interpretation could be interactive elements.

Transformation drive observations also appeared to relate to tempo regulation, expressive interpretation through the dynamic shaping of phrases, and technical elements. Certain transformation drive observations were seemingly influenced by technical elements, where the particular music score being enacted required certain reaching actions and movement to position the body and arms in order to strike the correct notes. Other transformation drive observations reportedly related to performer concentration, where the score indicated technically challenging aspects of the music calling for increased attention. Expressive bodily movement related to performer concentration is a new observation. Arguably, concentration and increased attention could display as reduced movement quantity. This idea might be analogous to Wanderley et al.'s (2005) observation of performers decreasing movement when playing technically challenging passages.

Although we did not observe the typical body sway movement reported in other studies (Clarke and Davidson, 1998; Williamon and Davidson, 2002; Davidson, 2007, 2012; Keller and Appel, 2010), the shaping features observed might reflect a type of this movement that is unique to marimba playing. Marimba playing typically involves a high degree of spatial movement on the horizontal, and to a degree, sagittal planes, using the feet to move the body around the instrument and position the body and arms in order to strike the required notes. As such, the prevalence of “Rising” and “Sinking” shape feature observations found in this case study might reflect an expressive body sway-type of movement somewhat special to marimba playing.

A possible interpretation of the patterns of effort-shape observations in this case study is that expressive movement might be hierarchically organized (Davidson and Correia, 2002; Davidson, 2005). Specifically, the observations of basic effort actions and shape features occurring separately and co-occurring might indicate hierarchic organization. As suggested by Davidson (1994, 2002, 2012), a body sway type of movement might operate as a center for expressive movement at a broad hierarchical level, with localized movements in various parts of the body operating at a more detailed level of the hierarchy. Of course, this proposition would require systematic investigation. A further area warranting systematic investigation concerns the driving forces for the expressive bodily movements generated. Previous research suggests that the music structure, technique, and expressive interpretation might be important elements to consider (Davidson, 2002, 2007; Wanderley, 2002; Thompson et al., 2005; Wanderley et al., 2005). Future research should investigate these elements and whether they are hierarchically organized and interactive.

Having identified a core repertoire of expressive marimba playing bodily movements in solo performance, we wonder if this might be evident in a collaborative music making context as well. Furthermore, we explore how the music-related features associated with the expressive bodily movements in a duo context compare to those identified in solo performance. We also seek to explore how transformation drives, evocative of mood or emotion, might differ between the two case studies featuring pieces of different musical characters. With the results of Case Study 1 indicating that basic effort actions and shape features can co-occur as well as appear separately, we seek to examine this phenomenon as evidence toward the idea that expressive bodily movements might be hierarchically organized. Finally, we further investigate the notion that individual differences in observers' motor expertise might shape their detailed perception of expressive bodily movements in marimba performance, beyond broad categories of movement types, by involving two professional musicians with differing marimba-playing expertise.

## Case study 2: marimba performance in a duo context

### Participants

Participants in Case Study 2 were both professional musicians. Participants were a female percussionist and experienced marimba player (aged 29 years), and a classical vocalist (aged 49 years).

### Materials

The material for analysis was *Cinq Pantomimes Pour Flute et Marimba, IV* by Damase (2002, 1 min 33 s). The performance, featuring the first author (female) performing the duet with a flutist (female), was recorded audio-visually during a professional chamber music recital. The music was unfamiliar to the observer participants. A DVD of the performance recording was provided to participants for playback on computer or television.

The observer participants in Case Study 2 were asked to complete two self-report questionnaires to assess movement imagery ability, and interpersonal non-verbal sensitivity in relation to socio-emotional competency. These abilities were assessed as screening measures since effort-shape analysis relies on a sound ability to visually and kinesthetically mirror observed expressive action, and glean some understanding about another's internal state. Tests of these abilities were only introduced into the research design for observer participants in Case Study 2.

Case Study 2 participants completed the Movement Imagery Questionnaire—Revised^1^ (MIQ-R, Hall and Martin, 1997). The MIQ-R asks participants to perform a series of simple motor tasks, then imagine themselves either visually or kinesthetically performing each task and rate the ease with which they could perform the imagery task on a seven-point Likert scale. Scores are summed separately for the two subscales to give separate visual and kinesthetic imagery ability scores. The questionnaire comprises eight items; scores range from 4 to 28. Higher scores indicate greater imagistic ease or imagery ability. Case Study 2 participants both reported high and comparable movement imagery ability as measured using the MIQ-R (visual imagery subscale score—percussionist = 27, vocalist = 27; kinesthetic imagery subscale scores—percussionist = 25, vocalist = 26). Scores were well above average.

Participants in Case Study 2 also completed the MiniPONS^2^ (Bänziger et al., 2011), which is the short version of the Profile of Non-verbal Sensitivity (PONS—Rosenthal et al., 1979). The MiniPONS examines participant accuracy in decoding multimodal affective non-verbal cues. Participants are presented with 64, 2-s clips of a woman, who appears to be interacting with another individual, expressing different emotional qualities. The non-verbal clips are presented in audio-only, audio-visual, or visual-only formats. Participants are asked to select the best-fit situational explanation for the woman's non-verbal expression. Accuracy scores range from 0–64. Both participants in Case Study 2 self-reported comparable interpersonal non-verbal sensitivity as measured using the miniPONS: percussionist = 52 (81%), vocalist = 51 (80%). The scores were well above chance.

### Procedure

Participants gave informed written consent prior to taking part in the research. The research conformed to Australian regulatory standards. The observational research methodology was approved by The University of Western Sydney (now Western Sydney University) Human Research Ethics Committee. Additional approval was obtained from the University of Western Australia Human Research Ethics Officer when both authors were based at that institution.

### Observer training: case study 2

Both participants were provided a 1.5-hour individual training session in effort-shape analysis, and the particular analysis task. Again, the first author provided the training, as she was experienced in conducting effort-shape analysis as applied to music, and in particular marimba, performance. During the training session, participants were introduced to effort-shape analysis, written reference material and illustrative audio-visual examples were provided, and participants practiced the observational techniques and actions (see Broughton and Stevens, 2012; Broughton and Davidson, 2014). The audio-visual training examples, provided with annotated scores, as illustrations of the different types of actions were drawn from the results of a prior inter-judge reliability study for effort-shape analysis, presented here as Case Study 1 (Broughton and Stevens, 2012). Although the first author conducted the training session and was identified in the audio-visual performance recording for analysis, the participants only noticed her play the marimba in the recording for analysis. It is unlikely that priming effects occurred for two reasons: (i) the time-course of perceptual priming is typically very short (e.g., within an experimental session), with recent primes being more influential on judgments and priming effects dissipating quickly (Higgins et al., 1985; Hermans et al., 1994), and participants completed this task over a period of a week at their convenience; and (ii) the training materials were based on empirical data and presented in an unbiased manner to give equal weight to each component of the effort-shape system.

As in Case Study 1, participants considered the carriage of the body as a whole, focusing on limb, torso and head movement, as they deemed important to include in their analysis. Once participants were satisfied that they understood the effort-shape analytical system and felt confident that they could independently apply it to analyze the performance material, the analysis task and procedure were outlined. They were then given a period of ~1 week to complete the task at their own convenience. As in Case Study 1, participants were informed that they were able to start, stop, and replay the audio-visual recording as necessary, with and without sound, in order to complete the task.

### Case study 2 task

Following the training session, participants completed an independent analysis task. Participants were provided with a music score matching the audio-visual performance recording. The task asked of participants was to conduct effort-shape analyses of the performers' bodily movements in moments perceived as expressive in the performance recording. Participants were asked to document their effort-shape analyses on the music score and using annotation software for audio-visual material, *ELAN* (version 4.5.0, Max Planck Institute for Psycholinguistics; Lausberg and Sloetjes, 2009; Sassenberg et al., 2011). A detailed report of the procedure and task that resulted in the data used here as Case Study 2 is reported elsewhere (see Broughton and Davidson, 2014). Although the participants analyzed the bodily movements in moments perceived as expressive in the performance recording of both performers, only the analyses of the marimbist are reported here.

### Results

Again, the frequency of effort and shape observations per participant were totaled (see Table 2). Analysis then involved pooling the observations where both participants agreed on observing the same type of action at the same location in the performance material.

**Figure 2:**
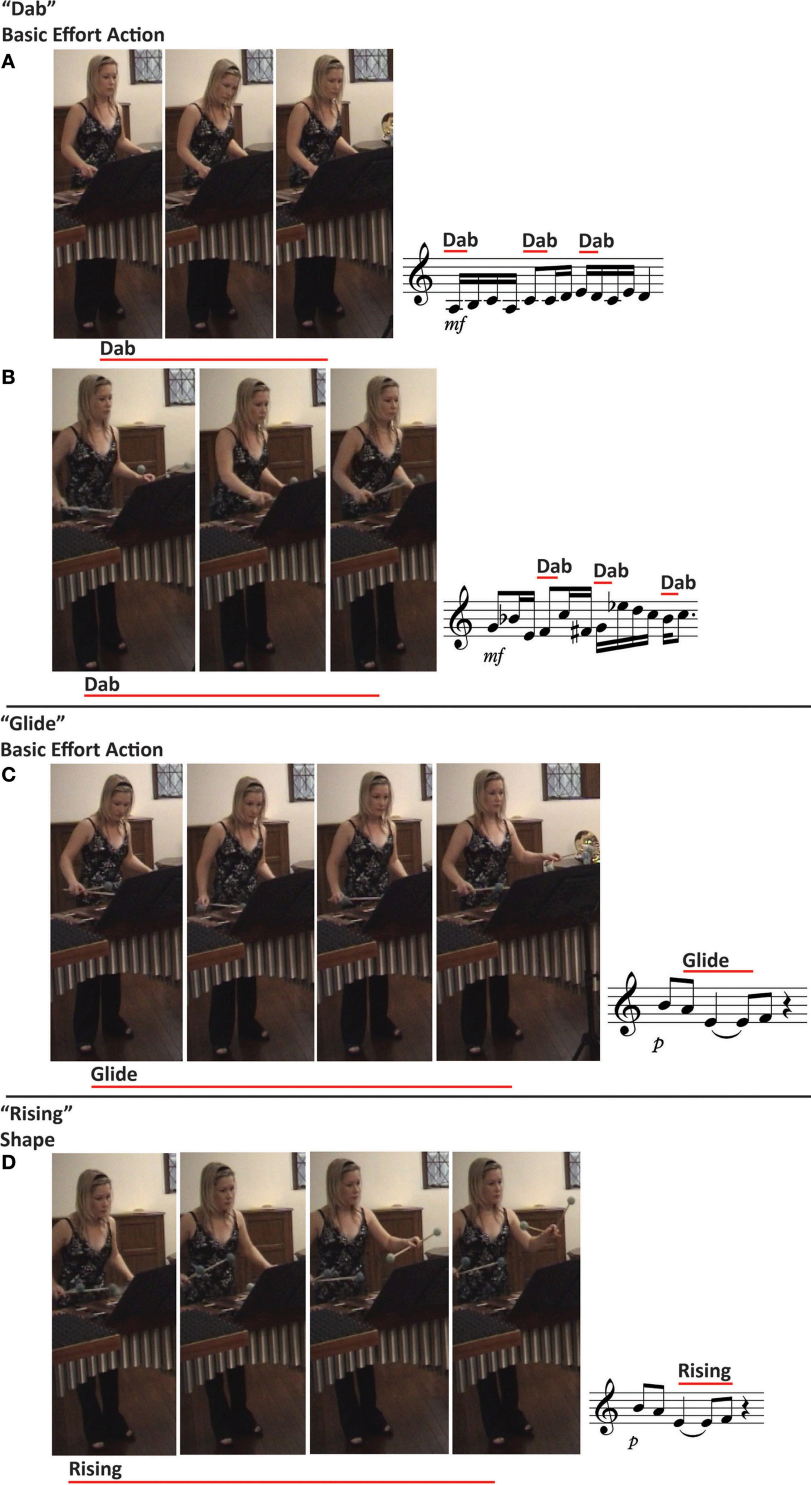
**Effort and shape feature movement examples as illustrated with still images drawn from Case Study 2**. The red line on top of the accompanying musical example illustrates the duration and placement of the movement in relation to the music performed. The number of still images included does not reflect the duration of the movement, simply sufficient images to illustrate each action type. The musical notation accompanying each example is indicative of the allied music excerpt, though not a reproduction from the original music score. Participants analyzed the carriage of the body as a whole, focusing on limb, torso, and head movement, as they deemed important to include in their analysis.

The percussionist and vocalist observed a small repertoire of expressive bodily movements. “Dab” (*n* = 10) actions were the primary expressive bodily movements observed by both the percussionist and the vocalist. “Dab” movements appeared to be allied to marking the tempo in rhythmic sections of the music played at a fast tempo, See Figures 2A,B. “Rising” (*n* = 3) shape features were observed both at the ends and peaks of phrases. They were also observed in conjunction with “Glide” (*n* = 1) basic effort actions. Figure 2 illustrates some exemplar expressive actions and indicative allied music excerpts (though not original reproductions from the music scores) for Case Study 2. Interestingly, at 15 locations in the performance material the percussionist reported observing “Float” basic effort actions where the vocalist reported “Rising” shape features. The percussionist and vocalist reported “Glide” basic effort actions accompanying long-duration notes in slow, lyrical sections of the performance material. Too few transformation drives were observed to draw meaningful conclusions. No “Slash” or “Wring” basic effort actions were observed.

**Figure 3:**
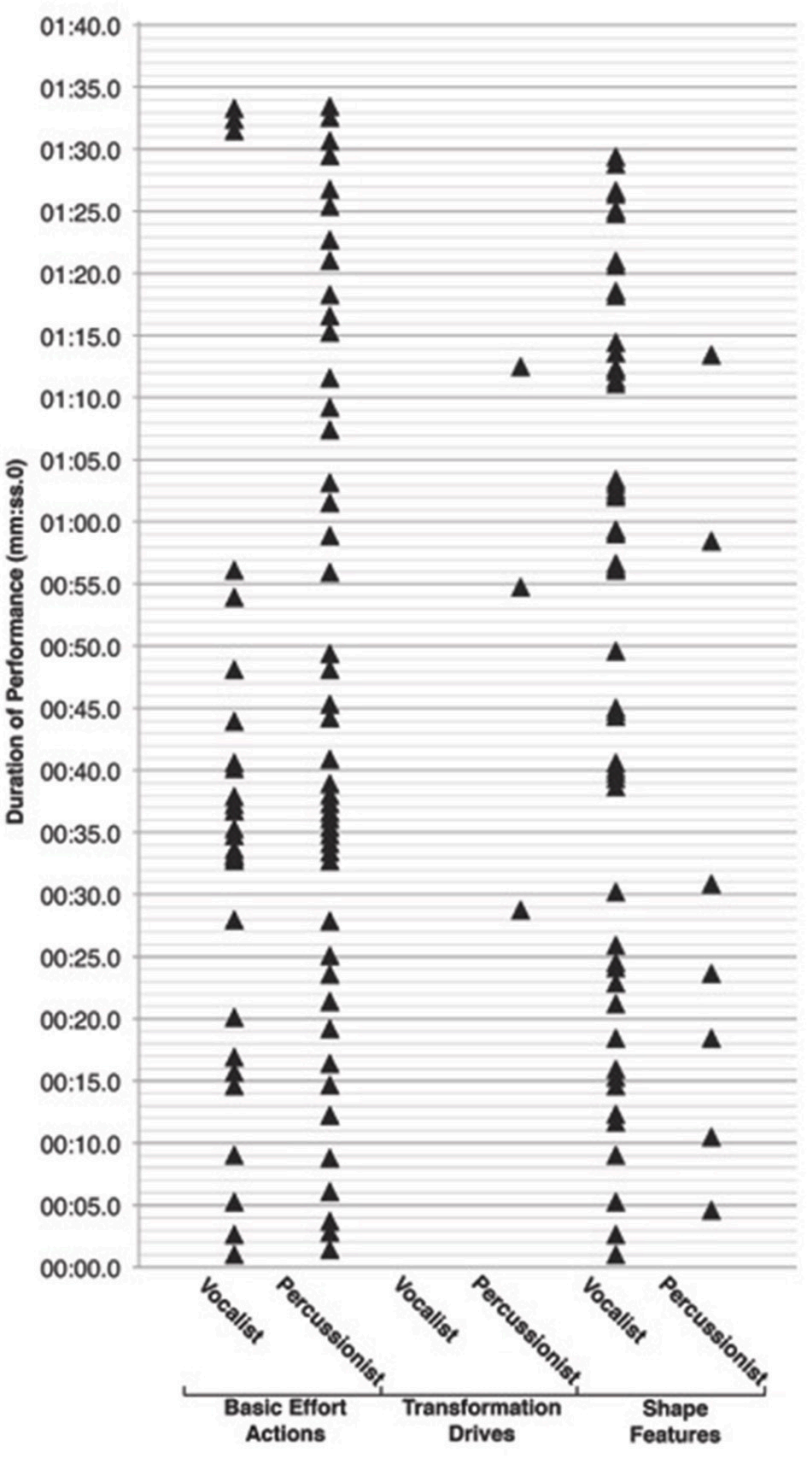
**Case Study 2 participants' effort-shape observations across the duration of the performance material**. The minor gridlines show the 2 s bins that were examined for agreement between the vocalist and percussionist for the same type of action in each category (basic effort actions, transformation drives, and shape features). Shared “Dab” observations (*n* = 10) reflect 31.24% of all “Dab” observations, and 13.16% of all basic effort action observations. Shared “Rising” observations (*n* = 3) reflect 7.14% of all “Rising” observations, and 5.08% of all shaping feature observations. The shared “Glide” observation (*n* = 1) reflects 7.14% of all “Glide” observations, and 1.32% of all basic effort action observations. Agreement results for observations of the same type of action at the same location in the performance material are reported in a previous publication (see Broughton and Davidson, 2014).

With the time stamped observational data from ELAN, we could create a graph to visualize the distribution of the percussionist and vocalists' effort-shape analyses across the duration of the performance material (see Figure 3). Looking at the distribution of the vocalist and percussionist's observations across the performance material, it is evident that basic effort actions and shape features co-occur (see Figure 3). However, there also are many more instances of basic effort actions and shape features being observed separately. For the percussionist, the frequency of basic effort actions observed alone (31, 65.96%), shape features observed alone (5, 10.64%), and basic effort action and shape features observed together (3, 6.38%) were significantly different, χ^2^(2, **n** = 39) = 37.54, *p* < 0.01. For the vocalist, the frequency of basic effort actions observed alone (9, 19.15%), shape features observed alone (17, 36.17%), and basic effort action and shape features observed together (10, 21.28%) were not significantly different, χ^2^(2, **n** = 36) = 3.17, *p* = 0.21.

Examination of individual participant data revealed differences in the frequency with which the various sub-components of the basic effort action, transformation drive and shaping feature categories were observed (Table 2). Separate chi-square goodness-of-fit tests conducted on individual participant data revealed significant differences between observational frequencies for different action types within the basic effort action, transformation drive, and shape feature categories.

Examining the results for the percussionist's observations, a significant difference in observational frequencies for basic effort action observations, χ^2^(3, *n* = 49) = 14.92, *p* < 0.01, was revealed with the order of most to least frequent observations being: “Dab” (18, 36.73%), “Float” (17, 34.69%), “Flick” (13, 26.53%), and “Glide” (1, 2.04%). Too few transformation drive or shape feature observations were made to conduct any analyses. For the vocalist, results were non-significant for basic effort action observations, χ^2^(1, **n** = 27) = 0.04, *p* = 0.85. The vocalist did not make any transformation drive observations, preventing analysis of this element of the system. A significant difference was found between observational frequencies for shape features, χ^2^(1, **n** = 52) = 11.08, *p* < 0.01. The order of most to least frequent observations were: “Rising” (38, 73.08%), then “Sinking” (14, 26.92%).

### Discussion

As in Case Study 1, a small repertoire of expressive bodily movements was perceived by the percussionist and vocalist at the same location in the performance material in Case Study 2. Contrary to Case Study 1, however, “Dab” basic effort actions were predominantly observed, and “Punch” actions were not reported. This is likely because the majority of the performance material being analyzed was of a lyrical style, at a slow tempo, and the fast rhythmic sections did not feature musical elements to be played in a strong and loud manner. The differences in expressive bodily behavior observations across the case studies might also reflect performer differences, or differences between solo and duo performance conditions. For example, the “Glide” basic effort action observed in Case Study 2 could reflect the flutist and marimba player aligning their expressive movement styles to a degree as a consequence of their interactions through the rehearsal process (Davidson, 2012). However, this idea is speculative, as the performance recording for analysis did not provide an opportunity to examine the marimba players' expressive bodily movement performing alone in comparison to performing with the flutist. Furthermore, the rehearsals were not recorded.

Common to both case studies, basic effort actions and shaping features were observed in relation to rhythmic note groupings, marking the fast tempo, and expressive phrasing of the music (Davidson, 2002, 2007; Wanderley, 2002; Wanderley et al., 2005). Once again, associations between the music score and expressive movement observations were not consistent. This suggests that additional concerns beyond the music score drive the production of expressive bodily movements. Transformation drives were rarely observed in Case Study 2. This might reflect differences in the musical characters of the music performed in Case Study 1 and Case Study 2. Alternatively, the additional perceptual and cognitive demands imposed by the need to coordinate with a co-performer could have meant that the musician was unable to direct her attention to this aspect of performance. The observed shape features, and perhaps even the “Glide” basic effort action observations might be similar to the body sway action observed in solo and collaborative music making contexts (Clarke and Davidson, 1998; Williamon and Davidson, 2002; Davidson, 2007, 2012; Keller and Appel, 2010). The results of Case Study 2 provide some support for the notion that shape features and basic effort actions co-occur as well as appear individually. As discussed in relation to Case Study 1, this might reflect the hierarchical organization of expressive bodily movement, with body sway, or “non-goal-directed” movement at a global level, and “goal-directed,” sound generating, actions at a local level (Davidson, 1994, 2002, 2005, 2012; Davidson and Correia, 2002). However, with evidence for this idea limited to a single case study, future research is required to investigate this notion systematically.

Some points in the performance stood out as shared moments of expression perceived by both the percussionist and vocalist, although categorized differently. For example, in certain instances the percussionist observed basic effort actions (e.g., “Float”) and the vocalist observed shape features (e.g., “Rising”). These moments suggest that the percussionist and vocalist were attending and responding to different facets of a shared expressive moment in the performance experience. As highlighted in Broughton and Davidson (2014), an observer sharing the same motor expertise for playing the instrument being observed might focus her/his attention on expressive movements necessary for sound production. In contrast, an observer with comparable music expertise but not motor expertise might perceive performers' expressive bodily movements in a slightly different manner, possibly influenced by her/his own embodied expertise.

## General discussion

The present study investigated the expressive bodily movements of marimba players through two observational case studies, using Laban effort-shape analysis. The first two aims of the present study were to: (i) investigate the existence of a core repertoire of marimba performance bodily movements that is perceived similarly by different observers as expressive, and; (ii) identify key music-related features associated with the core repertoire. The analyses of the two case studies revealed a small repertoire of expressive bodily movements seemingly characteristic of marimba playing. Although LMA is a refined approach to bodily movement analysis, the six observers who participated were able to learn it to a degree that they could apply it across different tasks and performance materials, with results revealing consensus on certain types of bodily movements that are core to expressive marimba playing. The identification of an expressive movement repertoire for marimba playing is consistent with previous studies observing expressive movement repertoires for other instruments (Clarke and Davidson, 1998; Wanderley, 2002; Wanderley et al., 2005; Davidson, 2007, 2012). The percussionists, violinist, and French hornist in Case Study 1, and percussionist and vocalist in Case Study 2 all reported witnessing “Dab” basic effort actions, and “Rising” shape features at similar locations in the respective performance material analyzed. “Dab” actions were allied to features of the score, such as phrase peaks (Davidson, 2012), rhythmic note groupings (Wanderley et al., 2005), and marking the tempo (Wanderley, 2002).

While “Dab” basic effort actions were noted predominantly in Case Study 2, “Punch” actions were most reported by the four observers in Case Study 1. The differences between the two case studies illustrates how performers could draw an appropriate movement from their core repertoire according to the characteristic of a particular music score and performance demands (Davidson, 2007). The selection of an expressive bodily movement from the repertoire appeared to be driven by several, potentially hierarchically organized and interactive, elements. These elements included music structure, technique, and expressive musical interpretation (Davidson, 2002, 2007; Wanderley, 2002; Thompson et al., 2005; Wanderley et al., 2005). How these elements interact is arguably highly individualistic, and dependent on the music score enacted.

“Rising” shape features were observed in conjunction with “Dab,” “Float,” and “Punch” basic effort actions in Case Study 1, and “Glide” basic effort actions in Case Study 2. Thus, “Rising” shape features could be viewed as an expressive movement on their own, or the consequence of movement required for sound production. Others making this distinction refer to ancillary (expressive) movements and instrumental actions (Wanderley et al., 2005; Nusseck and Wanderley, 2009). Looking at the relationship between basic effort actions and shape features, the results of the present study suggest that while ancillary movements and instrumental actions can occur separately, equally as much they appear as tightly coupled, or integrated actions. The observation in Case Study 2 that “goal-directed” basic effort action and shape features, reflecting postural effort, could co-occur or occur separately might suggest that expressive bodily movement is hierarchically organized (Davidson, 1994, 2002, 2005, 2012; Davidson and Correia, 2002). That is, postural shaping movements operate at a global level and “goal-directed” basic effort actions at a local level. Although across the two case studies we found no evidence for a typical body swaying observed in other studies of expressive performance (Clarke and Davidson, 1998; Williamon and Davidson, 2002; Davidson, 2007, 2012; Keller and Appel, 2010), the shaping features reported by participants might reflect a type of body sway movement. That is, “Rising” shape features might reflect a pattern of body sway-type movement that is unique to marimba playing. This expressive movement on the vertical plane could be because the practicalities of marimba playing requires bodily movement on the horizontal, and to a degree sagittal, planes, sometimes across great spatial distances and into awkward body positions to play the notes required by the music. Furthermore, as a performer is not in direct tactile contact with the instrument, vision and proprioception are important to hitting the right notes. Swaying in a circular motion, or moving on the horizontal or sagittal planes would likely challenge effective feedback from these perceptual systems and motor planning, to a degree dependent on the difficulty of the music performed, and result in note inaccuracies. Therefore, the vertical plane might afford expressive bodily movement more so than other spatial planes.

Interestingly, “Sinking” shaping features were not as perceptually salient for observers. Attention seemed to be directed to the “Rising” portion of vertical movement rather than “Sinking.” Perhaps the tension inherent in a “Rising” body posture captured observers' attention more than the sense of relaxation characteristic of a “Sinking” posture. “Rising” demands more muscular energy to perform than “Sinking,” as the movement requires the performer to counteract external forces. By contrast, “Sinking” involves yielding to external forces. From a human movement perspective, postural stability and voluntary postural movement requires the muscles, as internal forces, to oppose the external forces of gravity, and the ground (Bouisset and Do, 2008). In analyzing shape, “Rising” might have been the more salient feature, recruiting more sensory-motor resources to perform than “Sinking.” Furthermore, Wallbott (1998) reports that movements and postural behavior can indicate the quality or intensity of different emotional states. These states can be broadly categorized as distinguishing “active” emotions, such as hot anger and elated joy featuring high movement activity from “passive” emotions such as, shame and sadness with characteristic low movement activity. High movement activity, requiring increased muscular energy and tension, may be initially more perceptually salient to observers, as an ability to readily identify and respond to tension perceived in another's actions is perhaps advantageous for a social species such as ours (see Atkinson et al., 2004). Too few transformation drives were observed across the case studies to draw meaningful conclusions. Hardly any transformation drives were observed in Case Study 2, possibly indicating that the music was not conducive to performing them, or they were simply not expressively salient to observers. The differences between the case studies might reflect differences between the perceptual and cognitive demands, and goals, in solo and collaborative music making contexts. However, this assertion would require systematic investigation. No “Slash” or “Wring” basic effort actions were observed in either case study, suggesting that these types of actions are not appropriate to marimba playing.

Expressive movement observations were not consistently related to markings in the music score. For example, there might be a series of accent markings in the score but only one being associated with an expressive movement observation. One possible explanation of this finding accords with Godøy's (2008, 2010, 2011, 2013) notion of “goal points”. The “chunking by goal-points” hypothesis suggests that salient musical and gestural units are delineated not by musical boundaries, but by the central goal point of the initiated action. These salient gestural units appear nested in higher-order musical units, such as the phrase. In the present study, chunking appeared to accord with the expressive intention, and not just the physical sound production goal point. This suggests that music structure boundaries might be less important than expressive interpretation, which can transcend boundaries, in generating expressive movement patterns. Or perhaps expressive interpretation, technique, and the music structure at the higher hierarchical level of the phrase are interactive elements, and subsume lower-level elements of the music structure, such as accents and note groupings. As a further exploration of this idea, consider the “Dab” expressive movements perceived in the center of the phrases, rather than at the boundaries.

The third aim of the study was to understand how observers' perception of expressive bodily movements in marimba performance might differ, according to observers' individual differences in music and motor expertise. Looking across both case studies we see points of convergence and divergence in participants' observations. The violinist (Case Study 1) and the vocalist (Case Study 2) favored “Glide” basic effort action observations more so than the other participants. The violinist also observed many “Widening” shape features. Furthermore, the vocalist observed “Rising” shape features where at the same location the percussionist (Case Study 2) observed “Float” basic effort actions. Such observations might reflect a perception-action effect. The degree to which an observer can anticipate, or predict the result of the action of another is shaped by the observer's own motor expertise relative to the action that is being observed (Aglioti et al., 2008). In a study of elite basketball playing, Aglioti et al. (2008) report that motor experts can draw critical information regarding the effect of an action from early kinematic cues arising from the body movement of the person performing the action, as the action is being prepared. In a music context, Wöllner and Cañal-Bruland (2010) report that string players exhibited greater accuracy and timing consistency than non-string-playing musicians and non-musicians in a visual perception task requiring participants indicate the entry of the music from the leading movement of the first violinist in a string quartet. In the present study, we found further evidence to suggest that individual motor expertise might shape perceptual processing of audio-visual performance material. The percussionists and French hornist in Case Study 1 reported “Press” basic effort actions and many “Sinking” shape features. The percussionists and French hornist (Case Study 1), and percussionist (Case Study 2) observed many “Float” basic effort actions. The percussionist (Case Study 2) also reported many “Flick” basic effort actions. The variety in individual observations possibly indicates differences between participants' music and specific motor expertise, attention to different aspects of the performance material, as well as the music and performance context (Broughton and Stevens, 2012; Broughton and Davidson, 2014). The variety in observations between the different participants possibly also reflects sensory attenuation effects.

We have previously argued that planning or executing observed expressive movements in the analysis process might lead to sensory attenuation effects (Broughton and Davidson, 2014). That is, where the sensory effects anticipated for planned actions match the actual sensory inputs, this can weaken perception of the real sensory inputs (Wolpert, 1997). As a practical example, it is difficult to tickle oneself (Blakemore et al., 2000). Therefore, when an individual observer focused their attention on a particular aspect of the analytical system, such as “Rising” shape features, the observer's perception of expressive bodily movement might have been attenuated as a consequence of planning and executing the observed movements through the analytical process. Sensory attenuation might have impacted on the observer's perception of any further expressivity in that moment. As a result, the observer might have concluded that they had analyzed all the expressivity possible in the initial “Rising” and not attended to the “Sinking” part of the action. As further explanation, the act of coordinating, or synchronizing action with events in the environment involves interactive processes of adaptation and anticipation (van der Steen and Keller, 2013). Internal models about body-environment relationships can be inverse or forward (Wolpert et al., 1995). Inverse models use incoming sensory information to generate a motor command and effect a change in state. Forward models are predictive in that they allow for anticipation of the effects of a motor command on the environment or body. While these two models are interactive in action experience, execution or observation (Wolpert and Kawato, 1998), action anticipation can result in sensory attenuation effects. Future research will examine the potential effects that might result from the different processes in which observers engage as they conduct effort-shape analysis. In addition, it is recognized that differences between observers' perception of expressive bodily movement may also indicate that the training for the analytical system requires further refinement.

From this study, several directions for future research have emerged. First, it is crucial to develop a better understanding of the potential hierarchical organization of elements driving the production and perception of expressive bodily movements in music performance. The “goal points” hypothesis in relation to the generation and perception of expressive performance offers an interesting perspective from which to investigate how performers and observers segment expressive movement and musical material meaningfully. Key limitations to drawing firm conclusions from the results of this study relate to the small number of observers involved and performance cases analyzed. In addition, comparisons between solo and duo contexts are limited to the one solo and one duo case analyzed. Further research with additional case studies, including different instrumental and vocal musicians performing a variety of different musical works, and with additional observers conducting analyses will help strengthen the results found here and the validity of the inter-subjective effort-shape analytical system. Furthermore, future work will involve non-musicians as observer analyst participants for a point of comparison to the perceptual data provided by the music, and music and motor expert groups. As the six participants were permitted to analyze the performance material with and without sound, the relative contribution that auditory and visual information might have made to their analyses is yet to be examined. Although we expected that the musically trained participants' individual effort-shape analyses would not differ, whether analyzed with or without sound (Davidson, 1993), future research will investigate the different ways in which observes might conduct their analyses through diarizing and experimental methods. Finally, it is unknown how the task of conducting effort-shape analysis might perturb the naturalistic observations musicians, teachers, or audience members have of expressive bodily movement in performance material. Future work will conjoin experimental and observational approaches in an effort to address this research question.

## Conclusions and implications

The results of the present study revealed a core repertoire of bodily movements generated in expressive marimba playing that is perceived as expressive by observers. Across the two case studies, “Dab” basic effort actions and “Rising” shape features were observed. However, the individual case studies featured a slightly wider variety of characteristically different bodily movements that were perceived as expressive. While relationships between expressive bodily movements and certain music-related features were found, these were not always consistent. Individual performers seemed to draw on their core repertoire according to the character and demands of the music and performance context. Individual differences in observers' music and motor expertise appeared to shape their attention to and perceptual processing of expressive bodily movements.

Further research is necessary to understand more fully the organization and interplay of elements contributing to bodily movement in the production and effective communication of expressive music performance. With a common movement meta-language, such as the effort-shape framework, future research can examine in detail the nexus of performance-based and performance-led approaches to understanding musicians' expressive bodily movements. In doing so, it may be that this type of study could lay the foundation for programs for embodied expressive performance training. Such programs could involve training for deliberate development of an expressive movement plan for a music score in light of understandings about observer perception of expressive bodily movements for the particular instrument. Such evidence-based performance training programs might contribute to enhanced expressive communication for the mutual benefit of performers and audience members alike.

## Author contributions

MCB was primarily responsible for the study design, data acquisition, analysis, interpretation, and drafting and revising the manuscript. JWD contributed intellectually to the study design, and was involved in data acquisition, interpretation, and manuscript revision.

### Conflict of interest statement

The authors declare that the research was conducted in the absence of any commercial or financial relationships that could be construed as a potential conflict of interest.

## Footnotes

^1^The MIQ-R is a reliable and valid test of movement imagery ability: Test-retest reliability (0.80 visual subscale, 0.81 kinesthetic subscale—Monsma et al., 2009), internal consistency (0.79 visual and kinesthetic subscales—Vadoa et al., 1997; Monsma et al., 2009), bi-factorial structure (Lorant and Nicholas, 2004; Monsma et al., 2009).

^2^The MiniPONS is a reliable and valid test of interpersonal nonverbal sensitivity in relation to socio-emotional ability (Bänziger et al., 2011): correlation with the full PONS (*r* = 0.70), internal consistency (intraclass correlation coefficients—single item = 0.021; combined items = 0.566), test-retest reliability for the 64 items (*r* = 0.64).
